# Exploring L2 Engagement: A Large-Scale Survey of Secondary School Students

**DOI:** 10.3389/fpsyg.2022.868825

**Published:** 2022-06-14

**Authors:** Jing Wang, Bin Ying, Zhixin Liu, Rining Wei

**Affiliations:** ^1^School of Foreign Languages, Nanjing Xiaozhuang University, Nanjing, China; ^2^High School Affiliated to Southwest University, Chongqing, China; ^3^Department of Applied Linguistics, Xi'an Jiaotong-Liverpool University, Suzhou, China

**Keywords:** L2 proficiency, L2 engagement, psychological profile, parental attention, study time, parental coaching frequency

## Abstract

Engagement, a psychological individual difference variable with three facets (vigour, dedication and absorption), has recently attracted scholarly attention. Through a large-scale survey, we examined what we call ‘L2 engagement’ among 21,370 secondary school students in China, with an L2 engagement scale adapted from the Utrecht Work Engagement Scale (UWES)-student version. Factor analysis showed this scale to be empirically unidimensional with three highly intercorrelated facets and very high internal consistency; this contributes to our understanding of the conceptual challenges surrounding the construct of engagement (e.g., dimensionality) and the broader issue concerning the correspondence between empirical constructs and theoretical terms (e.g., engagement in our case). Hierarchical regression revealed that the selected sociobiographical variables (e.g., L2 proficiency) were linked to L2 engagement to varying degrees; adopting a more refined approach to gauge the unique contribution of a predictor to L2 engagement in hierarchical regression, we identified L2 proficiency, parental attention, study time and frequency of parental coaching as (very) important predictors for L2 engagement. We call for more studies to adopt our L2 engagement scale, a sufficiently valid and reliable instrument developed based on a large sample. We also propose a few future research directions (e.g., combining self-reports with other data sources).

## Introduction

Individual differences (IDs) on the part of the student have been a key area in applied linguistics research (Gardner, 1985; Dörnyei and Skehan, 2003; Dörnyei and Ryan, 2015). The focal IDs can be divided into two broad categories: cognitive and non-cognitive (e.g., psychological) ones (Wei and Hu, 2019; Luo and Wei, 2021). Psychological IDs (e.g., Dewaele and Li, 2013) remain a much under-investigated subcategory of IDs compared with cognitive ones (e.g., working memory, see Wen and Li, 2019).

In the field of applied linguistics, two distinct lines of research concerning psychological IDs can be identified (Wei et al., 2020): (1) the research tradition treating psychological IDs as the independent variables and L2 variables (e.g., target language achievement) as the dependent ones (see Schumann, 1976, 1986; Baker, 1996) (2) an emerging research line treating psychological IDs as the dependent variables (Dewaele and van Oudenhoven, 2009; Dewaele, 2012; Wei et al., 2020; Luo and Wei, 2021). Both types of research are valuable because they provide useful information concerning the psychological profiles of L2 learners (Wei et al., 2020; Wei and Gao, 2022) and complement the rich ongoing research on cognitive IDs (e.g., Dewaele and Li, 2013; Wei and Hu, 2019) in language learning. The present study, focussing on L2 learning engagement, contributes to the second line of inquiry.

Engagement is a positive, fulfilling and work-related psychological construct that is believed to comprise three facets: vigour, dedication and absorption (Schaufeli et al., 2002). One commonly used instrument to evaluate this construct is the Utrecht Work Engagement Scale (UWES). When it comes to students, the UWES has a student version (Schaufeli and Bakker, 2003). A student’s academic engagement has been suggested to be crucial for student learning in general (e.g., Skinner et al., 1990) and L2 learning in particular (e.g., Ibrahim, 2016). However, this psychological ID has been under-examined in applied linguistics research; while there have been a few (primarily) quantitative studies pertaining to engagement (e.g., Aubrey, 2017), the sample sizes of most studies have been relatively small (e.g., Nakamura et al., 2020). There remains a need for studies based on larger samples along this research line, because a larger sample tends to have less sampling error and higher representativeness of the study population (Loewen and Plonsky, 2016). Responding to this need, the present study makes the first attempt to examine engagement^1^ in L2 learning through a large-scale survey.

Besides contributing to the emerging research line mentioned in Paragraph 2, the significance of the present study can be found in three further areas. Firstly, our focal ID (*viz.* a positive psychological variable) falls within the category of ‘relatively unexamined constructs’ (MacIntyre et al., 2019, p. 262) that are only beginning to receive research attention since the introduction of positive psychology into applied linguistics research in the early 2010s (Dewaele et al., 2019), albeit negative psychological IDs (e.g., anxiety, see McEown and Sugita-McEown, 2020) have been very much well-researched. Against the backdrop of a ‘positive turn’ over the past two decades in applied linguistics research (Dewaele et al., 2019; Xu, 2021), the present exploratory study is timely not only because our focal variable is a positive psychological ID but also because it is one important building block for a L2 learner’s wellbeing [e.g., see the PERMA model in Seligman (2018)]. Secondly, our study (especially the part concerning the dimensionality) helps address Mercer’s (2019, p. 646) theoretical question on ‘the exact nature’ of our focal variable. Thirdly, the present study increases our understanding of the psychological profiles of multilinguals in China, an ‘under-investigated’ L2 context (Wei and Hu, 2019), where the number of English-knowing Chinese multilinguals already exceeded 417 million in 2015 (Wei and Su, 2015). This sheer number may merit some research priority.

## Literature Review

### Student Engagement

Student engagement has been variously referred to as ‘learner’ or ‘school’ engagement (Finn and Zimmer, 2012). According to Mosher and MacGowan’s (1985, p. 14) classic definition, engagement is ‘the attitude leading to, and the behaviour of, participation in’ the education institution’s programmes. More recently, Skinner et al. (2009, p. 494) define this construct as ‘the quality of a student’s connection or involvement with the endeavour of schooling and hence with the people, activities, goals, values and place that compose it.’

There has been long on theoretical debates but short on empirical evidence concerning the dimensionality of engagement. Some researchers argue for the *unidimensional* structure (Johnson et al., 2001; Skinner et al., 2009; Meng and Jin, 2017; Tsao et al., 2021), two (Green et al., 2007; Brutt-Griffler and Jang, 2019), three (Wang and Degol, 2014) or even four dimensions (Fredericks et al., 2016; Philp and Duchesne, 2016; Wang et al., 2016). For example, Green et al. (2007) conceptualised engagement as a bidimensional construct with one affective (i.e., positive attitudes towards school) and one behavioural (i.e., absenteeism, homework completion and class participation) component. On top of a three-dimensional structure (affective, behavioural and cognitive), Wang et al. (2016) proposed an additional social dimension, which purportedly could reflect the quality of social interactions with peers and adults while learning. Readers are reminded that the empirical studies to be reviewed below, more often than not, operationalise engagement in different ways (Reschly and Christenson, 2012), which reflects the conceptual challenges surrounding engagement.

In the past 30 years, a myriad of studies have documented the positive associations between student engagement and academic achievement (Finn and Voelkl, 1993; Connell et al., 1994; Philp and Duchesne, 2016; Wang et al., 2016).^2^ To our best knowledge, Skinner et al. (1990) is probably the earliest widely cited quantitative study on student engagement and school achievement. It confirmed the link between students’ academic achievement in two subjects and their engagement among a sample of 200 students (aged 9–12) at one suburban elementary school. In this study, the students’ engagement was measured through a 10-item scale scored by their teachers. One major finding was that the strength of the association (*r* = 0.41, *p* < 0.001) between student engagement and reading score reached the very strong benchmark (0.30) in the effect size interpretation system adopted in the present study,^3^ which was similar to that between engagement and maths score (*r* = 0.40, *p* < 0.001).

Voelkl’s (1997) methodologically strong study examined the relationship between student engagement (with two distinct theoretical dimensions: affective and behavioural) and academic achievement among 1,335 eighth graders. Affective engagement correlated weakly with the fourth-grade achievement (*r* = 0.07, *p* < 0.05) and was linked to the seventh-grade achievement at a typical level (*r* = 0.10, *p* < 0.01). In contrast, behavioural engagement very strongly correlated, respectively, with the fourth-grade achievement (*r* = 0.42, *p* < 0.01) and seventh-grade achievement (*r* = 0.49, *p* < 0.01).

A most recent quantitative study by Martin et al. (2020) demonstrated the correlations of students’ engagement, respectively, with age, socio-economic status (SES) and language background among 2,803 teenagers (average age: 14.14) at six Australian high schools. For science subject-specific engagement, these authors identified two types: positive and negative. Their path analysis revealed that (1) age (*β* = −0.16) and SES (*β* = −0.06) statistically significantly correlated with positive engagement and (2) non-English speaking background statistically significantly correlated with negative engagement (*β* = 0.03). The latter result is of particular interest to us because it pointed to the possible link between L2 proficiency and engagement. Unfortunately, while Martin et al. (2020) reported βs for the focal (independent) variables, the effect size index *β* is inconducive to comparisons across different studies; in contrast, Δ*R*^2^ is much more useful in cross-study comparisons.

### Student Engagement in Applied Linguistics Research

In the field of applied linguistics, only a limited number of empirical studies have investigated the complex construct of engagement, which can be divided into two subcategories. As Philp and Duchesne (2016, p. 385) rightfully note, engagement is often ‘not theorised or operationalised specifically for language learning’, which is similar to the situation outside of the field of applied linguistics (*cf.* Reschly and Christenson, 2012).

One subcategory of the extant research on engagement comprises studies focussing on more macro levels, such as the school subject level (*viz.* teaching L2 as a holistic school subject, see DeWaelsche, 2015) and the school level (see Archambault et al., 2009). For example, Brutt-Griffler and Jang (2019) discussed the relationship between L2 (English) proficiency and engagement among 53 Grade 6 students in New York, most of whom immigrated from Spanish-dominant societies (e.g., Puerto Rico) into the US. L2 proficiency was evaluated with a student’s self-rated proficiency. They claimed that L2 proficiency ‘was not correlated with’ behavioural engagement (p. 17), although the correlation coefficient was 0.26. This biased conclusion was largely attributed to the over-reliance on the value of *p*, as can be reflected in many instances of Brutt-Griffler and Jang’s (2019) paper where only statistically significant results were deemed important and discussed. However, effect size is much more important than the value of *p* (Wei et al., 2019; Wei and Gao, 2022).

According to the benchmark system cited above, the effect size *r* 0.26 was close to the very large benchmark (0.30) and hence should not be deeded as unimportant. The lack of statistical significance in Brutt-Griffler and Jang’s (2019) results was probably due to the relatively small sample size (*n* = 30) in their correlation analysis. Despite this limitation, Brutt-Griffler and Jang’s (2019) findings provided correlation coefficients (*viz.* effect sizes) for future studies to compare and contrast.

The other subcategory consists of studies focussing on more micro levels (e.g., at the mathematics classroom level, see Mesa and Chang, 2010; at the L2 classroom level, see Yu et al., 2019; at the knowledge construction level,^4^ see Berthoud and Gajo, 2020; at the instructional task-design level, see Lambert and Zhang, 2019; Sert and Amri, 2021). For instance, Aubrey et al. (2020) reported on a longitudinal study exploring the factors contributing to learners’ (lack of) engagement in an EFL classroom at one Japanese university; based on qualitative self-reported reflection data from 37 second-year sociology, they found that a lack of engagement could be attributed to causes, such as ‘lack of vocabulary’ and being unable to say what the student wanted to ‘say quickly in English’ (p. 8). In other words, L2 proficiency level could affect engagement.

Bai et al. (2020) investigated the relations between behavioural engagement and three motivational variables (academic self-efficacy, task importance and interest) among 1,954 secondary school English learners of the same grade level (i.e., the 9th grade) in Singapore. The self-reported behavioural engagement level was evaluated *via* a five-point Likert scale comprising four items. Their multivariate regression analyses showed that: (1) each of these three motivational variables statistically significantly (*p* < 0.001) predicted students’ behavioural engagement; and (2) these three motivational variables together accounted for 17.9% in the behavioural engagement variance. However, no attempts were made to ascertain the unique contribution of each predictor in the regression model.

All in all, the above-reviewed studies suffer from at least two of following three limitations: (1) the sample representativeness (e.g., Meng and Jin, 2017; Bai et al., 2020), (2) limited use of effect size (e.g., Brutt-Griffler and Jang, 2019; Yu et al., 2019) and (3) the over-reliance^5^ on the statistical significance level, namely, the value of *p* (e.g., Brutt-Griffler and Jang, 2019; Bai et al., 2020). Accordingly, the present study attempts to overcome the above limitations by covering different grade levels, making full use of effect size and avoiding over-reliance on *p*s.

### L2 Engagement

In connection with academic fields other than applied linguistics (e.g., personality psychology), Reschly and Christenson (2012) note that engagement still suffers from a jingle (i.e., different terms being used to refer to identical notions or constructs) and jangle (i.e., the same terminology being used to describe distinct notions and constructs) in the way it is defined and operationalised. In applied linguistics research, this state of affairs is no different (Hiver et al., 2021). In their most recent synthesis entitled ‘Measuring L2 Engagement: A Review of Issues and Applications,’ Zhou et al. (2020) do not propose a definition of ‘L2 engagement’, let alone its measurement. Another limitation is that a few recent studies (e.g., Aubrey, 2017; Brutt-Griffler and Jang, 2019) from major journals in the field unfortunately escaped Zhou et al.’s (2020) attention. Notwithstanding these limitations, they highlight ‘the variety of operational definitions’ (p. 79) concerning engagement used across studies; and they also rightfully observe that ‘one researcher’s conceptualisation of cognitive engagement is used as another’s measurement of behavioural engagement’, which is indicative of the conceptual challenges surrounding engagement.

Against this backdrop, L2 engagement is defined as the type of engagement specifically relating to the domain of a student’s L2 learning. In the present study, a student’s L2 engagement is operationalised as his/her score gained on a L2 engagement scale adapted from the UWES-S. To the best of our knowledge, the present study represents the first attempt to quantitatively measure ‘L2 engagement’ in a large sample, aiming to shed light on the empirical dimensionality of this construct and explore potential factors affecting it.

## The Present Study

### Research Questions and Analytic Strategy

RQ1: What is the factorial structure of the L2 engagement scale?

RQ2: To what extent do the selected sociobiographical variables (e.g., L2 proficiency, parental attention and parental coaching frequency) predict L2 engagement?

RQ1 was addressed using both exploratory factor analysis (EFA) and confirmatory factor analysis (CFA), in addition to reliability analysis. Through the random sampling function of SPSS, the data set was divided into two halves, which were, respectively, subject to EFA and CFA to ascertain the construct validity of the L2 engagement scale. To gauge the scale’s reliability, Cronbach’s alpha (a measure of internal consistency) was used in that it is the most frequently used reliability index (Derrick, 2016).

The selected sociobiographical variables in RQ2 totalled 11 (see Table 1 for a complete list). RQ2 was addressed with hierarchical regression because it helps ascertain the unique contribution from each predictor variable (Leech et al., 2014; Kong and Wei, 2019) to the variance in our focal variable, L2 engagement. As the L2 engagement variance explained by each predictor may change depending on the order in which the predictor is entered into a regression model, the chosen order is crucial (Luo and Wei, 2021). Researchers may determine the order for predictor entry based on theoretical grounds, if well-established theories exist; but in the absence of such theoretical resources, researchers are advised to attempt all possible sequences and provide a range of effect sizes (rather than one single effect size) for each predictor, which represents a more refined approach to gauge the unique contribution of a predictor to the dependent variable in hierarchical regression (Wei et al., 2020). The latter practice was adopted below because of the exploratory nature of the present study.

**Table 1:** Participant profile (*N* = 21,157).

Variable	Range	Mean (SD)/% (frequency)
L2 engagement	1–7	3.94 (1.17)
Grade	1–6	3.53 (1.53)
Monthly family income	1–6	3.48 (1.15)
Father’s education qualification	1–5	2.55 (1.00)
L2 proficiency	1–5	2.86 (1.00)
Mother’s education qualification	1–5	2.37 (1.03)
Parental attention	1–5	3.60 (0.90)
Parental coaching frequency	1–5	1.87 (1.07)
Study time	1–5	1.80 (0.70)
Level of urbanisation	1–3	2.23 (0.84)
Being the only child (or not)	binary	25.9% (5482)
Female (Gender)	binary	55.1% (11647)

CFA was performed with Amos 23.0, whereas the other statistical procedures were run with SPSS 27.0. For sake of convenience, the conventional statistical significance cutoff level (*α* = 0.05, non-directional) was adopted. Exact values of *p* were reported, except that very small values of *p* were reported as *p* < 0.0005.

### Instrument

The instrument comprised two major parts: the L2 engagement scale and the sociobiographical information section. This scale was adapted from the Chinese version of UWES-S (Fang et al., 2008). The UWES scale has been widely used to evaluate engagement in different cultural contexts, including China (Meng and Jin, 2017), India (Rastogi et al., 2017), Japan (Tsubakita et al., 2017), Turkey (Çapri et al., 2017) and the USA (Mills et al., 2012). It has also been adapted to some languages other than English, such as Chinese (Fong and Ng, 2012), Dutch (Schaufeli and Bakker, 2003) and Spanish (Schaufeli and Bakker, 2003).

In order to specifically focus on L2 learning (English learning in our case) rather than general learning, we replaced reference to ‘learning’ with ‘English learning’ on the Chinese version of UWES-S. The scale measured L2 engagement with 17 items on a seven-point Likert scale, with a score of 1 indicating ‘never’ and a score of 7 ‘always’. A participant’s score on this scale was an average of the scores from these 17 items; the higher the score, the higher degree of L2 engagement of this participant.

The sociobiographical information section covers 11 variables (e.g., L2 proficiency), most of which were included in previous studies (e.g., Johnson et al., 2001; Aubrey et al., 2020); for instance, L2 proficiency was evaluated with a student’s self-rated proficiency in English, which is consistent with previous research (e.g., Brutt-Griffler and Jang, 2019). It is worth noting that we included four under-investigated^6^ variables. Two concern parental input: degree of parental attention to their children’s L2 learning and frequency of parental coaching for L2 learning; our measures of parental input were different from those adopted in the few extant studies, including Butler (2015) who measured the so-called parents’ ‘direct behaviour.’^7^ Each of them was measured by one five-point Likert scale item (a higher score indicating a higher degree/frequency).

The other two under-investigated variables are a respondent’s study time devoted to L2 learning and his/her status of being the only child of the family or not. The former was gauged by one five-point Likert scale item asking the participant’s study time spent on L2 learning every day, with a score of 1 indicating ‘less than one hour’, 2 ‘one to two hours’, 3 ‘three to four hours’, 4 ‘five to six hours’ and 5 ‘more than six hours’; the latter was measured with one binary variable: being the only child of the family or not. The inclusion of these four under-investigated variables helps broaden the research scope for the research line investigating student engagement with L2 learning.

### Participants

Among the valid sample of 21,157 Chinese students, 3,350 (68.4%) were studying at Grade Seven, 2,918 (13.8%) Grade Eight, 1905 (9.0%) Grade Nine, 6,409 (30.3%) Grade Ten, 5,268 (24.9%) Grade 11 and 1,307 (6.2%) Grade 12. Slightly over half of the sample (55.1%, *n* = 11,647) were females and the remaining 44.9% (*n* = 9,510) males. About a quarter of the students (25.9%) were the only child in their family, and the others (74.1%) were not. At the time of the survey, a quarter of the students (26.3%) lived in villages, another quarter (24.6%) in townships and approximately half (49.1%) in cities.

### Procedure

The survey instrument was an anonymous, open-access questionnaire on Wenjuanxing,^8^ a free China-based survey provider similar to SurveyMonkey.com. The draft questionnaire had undergone several rounds of revision and was piloted among a small group of secondary school students before the questionnaire was finalised. The final questionnaire was published on Wenjuanxing between March and May in 2020. This final version of the questionnaire was distributed to students at a total of over 200 secondary schools in 18 out of the 31 ‘provinces’, which covered all of the six administrative regions of mainland China: the Central (e.g., Jiangxi), East (e.g., Zhejiang), North (e.g., Hebei), Northeast (e.g., Jilin), Northwest (e.g., Tibet), South (e.g., Guangdong) and Southwest (e.g., Chongqing). Specifically speaking, at each of the sampled schools, the contact person (*viz.* the head teacher of an intact class or the English subject teacher) explained the purpose of this survey and promised anonymity to the students at the end of a class; the students gained access to the online questionnaire by scanning the bar code provided by the contact person.

## Findings

### RQ1. The Dimensionality of L2 Engagement

Before performing EFA to explore the factorial structure of L2 engagement, we checked the relevant assumptions. The factorability of the data set was confirmed through the KMO test (0.977) and Bartlett’s test of sphericity [*χ*^2^(136) = 170813.572, *p* < 0.0005]. Furthermore, the sample-size-to-variables ratio (608.8) was very high. These assumptions checking results showed that our data set was appropriate for factor analysis.

We selected maximum likelihood as the factor extraction method and the direct oblimin rotation in that we assumed the extracted factors to be correlated, which is typical ‘for naturalistic data and certainly for any data involving humans’ (Field, 2013, p. 644). Aiming to extract the most appropriate number of factors, we employed both the Kaiser criterion of using eigenvalues over 1 and the visual inspection of a scree plot. We adopted Field’s (2013) suggested cutoff point of 0.40 for factor loadings. Only one factor, which accounted for 66.024% of the L2 engagement variance, was extracted (see Appendix 1). To ascertain the dimensionality of the L2 engagement scale, CFA was employed to compared four competing models: Model 1 defined one primary factor only and tested whether L2 engagement can be regarded as a unidimensional construct without three facets; Models 2 and 3 (see Appendix 2), respectively, defined three correlated and uncorrelated primary factors which correspond to the three theoretical factors defined by Schaufeli and Bakker (2003); Model 4 (see Appendix 3) defined a higher-order model with three first-order facets and one second-order underlying factor (L2 engagement).

Consequently, the fit indices for these models (Table 2) revealed that only Models 3 and 4 had adequate fit to the data. When simply based on the fit indices, it can be seen that Models 3 and 4 yielded virtually the same degree of model fit. However, considering very strong intercorrelations (above 0.9, see Model 3 in Appendix 2 for details), we suggest that L2 engagement should be treated as a latent variable indicated by the three factors, or as a multi-facet construct. Put differently, the empirical construct of L2 engagement has been shown to be unidimensional with three facets with our large-sample data set.

**Table 2:** Model fit indices of UWES-S scale measuring L2 engagement.

Model	*χ^2^/df*	*p*	GFI	TLI	CFI	RMSEA
1 Single factor	106.927	<0.0005	0.849	0.913	0.924	0.102
2 Uncorrelated three factors	309.449	<0.0005	0.747	0.747	0.779	0.173
3 Correlated three factors	57.089856800463	<0.0005	0.9197480035705	0.954002882089862	0.960767164135471	0.0739201243457364
4 Second-order factor with three facets	57.089856800458	<0.0005	0.9197480035763	0.954002882089866	0.960767164135474	0.0739201243457334

Reliability analysis indicated that this unidimensional scale had very high internal consistency (Cronbach’s *α* = 0.974). In a word, our answer to RQ1 was that the L2 engagement scale has sufficiently high reliability and validity in the Chinese EFL context.

### RQ2. Sociobiographical Variables Affecting L2 Engagement

Before running hierarchical regression, we conducted two rounds of data checking. The first round was a preliminary analysis; it aimed to explore which of the initial 11 independent variables could be used as predictors in regression analysis because entering too many independent variables into a regression model violates the principle of parsimony (see Luo and Wei, 2021). Consistent with the good practice of pre-selecting independent variables based on effect size rather than *p* (e.g., Kong and Wei, 2019; Wei et al., 2020), only potential predictors with an effect size value exceeding the typical benchmark (e.g., *r* = 0.1) were used in later regression.

The preliminary analysis, in which a series of bivariate analyses were conducted, confirmed that the first eight variables in Table 3 were suitable for inclusion into later regression. Specifically, one independent-samples *t*-test confirmed a statistically significant difference of medium magnitude between participants who was the only child of his/her family and their counterparts (*r* = 0.127); the former (*M* = 4.09, SD = 1.16, *N* = 5,314) scored higher on L2 engagement than the latter (*M* = 3.88, SD = 1.07, *N* = 15,302); that is to say, it appears that if a participant was the only child of his/her family, (s)he tended to have a higher L2 engagement score. Regarding the other seven variables, each of them statistically significantly correlated with L2 engagement, with their Spearman correlation coefficients ranging between 0.117 (monthly family income) and 0.563 (L2 proficiency); parental attention had the third highest effect size (0.307), which already exceeded the ‘very strong’ benchmark (0.30), suggesting that the more parental attention a respondent could receive for his/her L2 learning, the higher his/her L2 engagement.

**Table 3:** Links between the 11 initial independent variables and L2 engagement.

Variable	*Effect size r*/*r*_s_ (*p*)
L2 proficiency	0.563
Study time	0.343
Parental attention	0.307
Parental coaching frequency	0.213
Father’s education qualification	0.151
Mother’s education qualification	0.149
Being only child or not	0.127
Monthly family income	0.117
*(Below are variables ineligible for later regression analyses)*	
Level of urbanisation	0.095
Grade	−0.090
Gender	0.083

*The corresponding p for each of the above effect size values fell below 0.0005*.

The second round of data checking aimed to ensure that the assumptions (e.g., normality and homoscedasticity) for regression were met, in order to echo recent calls for the good practice of assumption checking (Hu and Plonsky, 2019). For example, when checking for potential outliers, based on several rounds of casewise diagnostic analyses, about 750 cases, which had a standardised residual greater than 3 or smaller than−3, were deleted from the initial sample (*N* = 21,370). The revised sample size for later analysis was 20,616.

After the above two rounds of checking, we performed a series of hierarchical regression analyses. In each hierarchical regression, we entered each of the eight predictors, one by one, into each of the eight models (or ‘blocks’ as called in SPSS). As mentioned above in the analytical strategy, when the predictors are entered one by one into regression, the entry order is crucial. A total of eight predictors will generate 40,320 (8×7×6×5×4×3×2×1) possible sequences and hence up to 40,320 different scenarios; that is to say, for each predictor, there could be 40,320 different effect size values. Hence, providing a range of effect size values for each predictor is much more informative and comprehensive, although many studies employing hierarchical regression simply report one single effect size value. Two sets of important findings emerged from our hierarchical regression analyses.

The first set included (1) the eight predictors (see Model 8 in Table 4) statistically significantly (*p* < 0.0005) predicted L2 engagement and explained a total of 41.3% in the L2 engagement variance (adjusted *R*^2^ = 0.413) and (2) each of the other seven models statistically significantly (*p* < 0.0005) L2 engagement.

**Table 4:** Hierarchical regression predicting L2 engagement: model summary.

	Model 1 Parental attention	Model 2 Parental coaching	Model 3 Study time	Model 4 Being only child or not	Model 5 Income	Model 6 Mother’s education	Model 7 Father’s education	Model 8 L2 proficiency
*R^2^*	0.100	0.121	0.188	0.190	0.194	0.195	0.196	0.413
𝛥*R^2^*	0.100	0.019	0.065	0.001	0.003	0.002	0.001	0.217
𝛥*F*	2308.971	446.178	1651.364	28.731	87.884	38.949	13.725	7622.713

*For Models 1–8, the variable underneath ‘Model’ indicates that it was the newly added predictor in this particular model*.

The second set of important findings comprised the ranges of the effect size *𝛥R^2^* for each of the predictors: L2 proficiency (21.7–34.2%), study time (2.4–11.9%), parental attention (1.4–10.0%), parental coaching frequency (0.7–6.7%), mother’s educational qualification (0–2.8%), father’s educational qualification (0–2.6%), income (0.1–1.3%) and being only child or not (0–0.7%). It is worth noting that for the first two variables, their maximums in the effect size range went above the very large benchmark (9%), and their minimums exceeded the large benchmark (2%), suggesting that both L2 proficiency and study time were very important predictors for L2 engagement. Regarding parental attention, its effect size maximum also exceeded 9%, although its minimum was lower than 2%, meaning that parental attention could also be a very important predictor, similar to study time. In contrast, parental coaching frequency, mother’s educational qualification, father’s educational qualification, respectively, had a maximum effect size higher than the large benchmark, which indicated that they could be important predictors for L2 engagement. Regarding the last three predictors, their effect size minimums could drop to 0.1% or zero, which showed that they might exert negligible or nil effect on L2 engagement and hence were relatively unimportant.

Table 4 summarises the key information of one example from the 40,000+ hierarchical regression scenarios predicting L2 engagement. In this scenario, parental attention was entered into the first block, parental coaching frequency the second, study time the third, being one child or not the fourth, monthly family income the fifth, mother’s and father’s educational qualification the sixth and seventh, respectively, and finally L2 proficiency; each block statistically significantly (*p* < 0.0005) added to the prediction of L2 engagement. The *𝛥R^2^* column in Table 4 revealed the most important findings: (1) L2 proficiency and parental attention, respectively, explained 21.7% and 10.0% of the variance in L2 engagement, which exceeded the very large effect size benchmark (9%), (2) study time accounted for 6.5% of the L2 engagement variance, which was higher than the large effect size benchmark (2%), (3) frequency of parental coaching contributed 1.9% in the L2 engagement variance, which nearly met the large effect size benchmark and (4) the unique contribution to the L2 engagement variance, respectively, from family’s monthly income (0.3%), mother’s education (0.2%), father’s education (0.1%) and being only child or not (0.1%) fell below the small effect size benchmark (0.5%) and hence seemed negligible. All in all, in this hierarchical regression scenario, L2 proficiency, parental attention and study time turned out to be/emerged as particularly important in terms of predicting L2 engagement, and frequency of parental coaching was also important. In addition, the positive *β* values (see Appendix 4) indicated the corresponding positive links; for example, the positive *β* (0.132 in Model 8, Appendix 4) for parental attention indicated that the more attention a participant received from his/her parents, the higher L2 engagement level he/she had; similarly, the higher L2 proficiency that a student had, the more engaged (s)he was with regard to English learning; the same could also be said about the other important predictors (e.g., study time).

## Discussion

In connection with RQ1, the present study has confirmed that the L2 engagement scale has sufficiently high reliability and validity in the L2 context investigated. This finding is consistent with previous studies (e.g., Meng and Jin, 2017) that also showed the UWES-S to be highly reliable and valid, although they utilised much smaller samples and focussed on the general learning domain. It is useful for future studies to (partially) replicate this study in other L2 learning contexts, which could range from those similar to the present context (e.g., the Chinese context involving university students) to those that are drastically different (e.g., the CLIL context or the heritage language revival context). Such (partial) replications significantly contribute to not only a further understanding of the L2 engagement scale’s validity and reliability across different contexts, but also the broader debate on the correspondence between empirical constructs and theoretical terms (*cf.* Carmines and Zeller, 1974). Specifically, in our case, although the three ‘dimensions’ of L2 engagement (our focal ID) can be claimed/theorised to theoretically distinct, the data showed this ID to be an empirical construct that is unidimensional with three facets. This important result helps confirm Carmines and Zeller’s (1974) insight that theoretical terms do not necessarily have a one-to-one correspondence with empirical constructs, since they can be operationalised and measured in an almost infinite variety of ways.

Our answer to RQ2 was that the selected sociobiographical variables (e.g., L2 proficiency) were linked to L2 engagement to varying degrees. Besides confirming the link between an independent variable and L2 engagement, we contributed an effect size range to reflect the strength of the link. Three points merit attention. Firstly, L2 proficiency turned out to be a statistically significant predictor for L2 engagement, which was consistent with both qualitative (e.g., Aubrey et al., 2020)^9^ and quantitative results (Martin et al., 2020)^10^ from previous research. Secondly, in terms of effect size, L2 proficiency emerged as the most important predictor for L2 engagement, which cannot be directly compared with previous studies and hence requires verification in future studies employing comparable effect sizes. Although L2 proficiency is usually regarded as an output variable (e.g., a factor in Stage 4 of Gardner’s (1985) socio-educational model), it will in turn exert strong influence upon a learner’s IDs (e.g., L2 engagement) during the subsequent stage of L2 learning, due to the ‘highly dynamic’ nature of L2 learning process (Gardner, 1985, p. 149). Thirdly, two sociobiographical variables (parental coaching frequency and parental attention) emerged as important predictors for L2 engagement. This echoed previous studies (Gao, 2006; Butler, 2015; Stubbs and Maynard, 2017) highlighting the important role of parental input. The result may be attributed to the fact that parents in China, who tend to have ‘the strong sociocultural desire’ for their children to be successful in life (Tan, 2013, p. 53), are usually involved in their children’s L2 learning very closely (Gao, 2006); this is particularly true with parents in major cities. Indeed, parents’ attitudes and behaviours (e.g., coaching frequency) may significantly motivate students to become more engaged with L2 learning.

In connection with methodological concerns, when reporting the results of one example scenario in the section “Findings”, we advised readers to refer to the *𝛥R^2^* column in Table 4 for the most important findings; among them, one particularly interesting finding merits attention: the variance-accounted-for percentage (i.e., *𝛥R^2^*) of parental attention (10.0%) was higher than that of study time (6.5%). If researchers had conducted only one such hierarchical regression analysis, they might have come up with a biased conclusion that parental attention was more important than study time. This was contrary to the conclusion that overall speaking (*viz.* based on 40,000 + regression analyses) parental attention was *less* important than study time, which was derived from the more compressive information offered by the effect size ranges of these two predictors. This is one of the major benefits of our above suggestion that a range of Δ*R^2^* (rather than one single Δ*R^2^*) for each predictor in regression should be provided.

A final noteworthy point is that for some predictors, their effect sizes were generally inflated in bivariate analyses, compared with their counterparts from hierarchical regression. For example, for the variable ‘being one child or not’, its effect size from the bivariate analysis (*r* = 0.127 in Table 3, equivalent to 1.6% variance-accounted-for in a simple regression predicting L2 engagement) was much larger than any value from its effect size (Δ*R^2^*) range (0–0.7%) generated from multivariate regression analyses. Given the multivariate nature of L2 learning, multivariate analysis paints a much more accurate picture than bivariate analysis (e.g., *t*-test and correlation).

## Conclusion

Engagement in L2 learning has received increasing attention in recent years (Nakamura et al., 2020), but most studies have drawn upon relatively small samples and/or suffer from some limitations in data analysis (e.g., limited use of effect sizes). Through a large-scale survey, the present study has confirmed that the L2 engagement scale (Appendix 1) is empirically unidimensional, with sufficiently high sufficiently high reliability and validity. It has also found that L2 proficiency, parental attention, study time and parental coaching frequency were important predictors for L2 engagement; the latter three were previously under-investigated independent IDs, suggesting that it was very much worthwhile for our study to include these predictors.

Regarding theoretical contributions, first, the present study has established the empirical unidimensionality of L2 engagement, which further our understanding of the focal variable that has been subject to different theoretical perspectives. In our case, we adopt the perspective of Schaufeli et al. (2002) who theorise the three dimensions of vigour, dedication and absorption as *conceptually distinct*, but our data showed these dimensions are highly interrelated (i.e., *empirically indistinct*). This is similar to situations where other theoretical perspectives are adopted (see, e.g., Mercer, 2019). This degree of the correspondence between empirical constructs and theoretical terms has seldom been explicitly discussed in previous research (*cf.* Carmines and Zeller, 1974). Second, echoing Dewaele’s (2012) call for a shift in research focus by looking at psychological IDs as the dependent variables, the present study has shed light on the highly dynamic or ‘cyclical’ nature of L2 learning process (Baker, 1996, p. 107), This points to a need for more research to examine the ‘more dynamic interaction’ (Dörnyei and Ryan, 2015, p. 33) between psychological IDs (e.g., engagement) and L2 proficiency. Third, more studies, such as the present on a positive psychological ID, are important because the positive is as worthy of study as the negative (MacIntyre et al., 2019, p. 267), albeit research on psychological IDs has mainly focused on negative ones since the 1970s (Dewaele et al., 2019). Fourth, the present study has extended the current research scope by including four under-investigated independent variables, three of which turned out to be (very) important predictors for L2 engagement.

In connection with methodological contributions, four suggestions are proposed for future studies. First, we confirm the value of using a more refined data analysis approach based on hierarchical regression (i.e., providing a range of effect sizes for each predictor) and advocate this more refined approach in future research. Second, echoing the advocacy for ‘fuller use of effect sizes’ (Kong and Wei, 2019, p. 50), we propose that researchers provide two or more types of effect sizes to measure the effect of interest, in order to facilitate comparisons across studies. Third, the L2 engagement scale (Appendix 1) developed in the present study is a sufficiently valid and reliable instrument and hence can be fruitfully employed in similar student populations and possibly beyond. Fourth, we employed a large-scale survey based on a very large sample, resulting in ‘less sampling error and therefore greater validity and reliability of the analysis’ (Loewen and Plonsky, 2016, p. 173).

Our results also have some practical implications for home–school partnership. As shown above, parents’ attitudes and behaviours (e.g., coaching frequency) can significantly enhance their children’s L2 engagement, which echoes the result consistently found in much research of school effectiveness that ‘the more parents are engaged in the education of their children, the more likely their children are to succeed in the education system’ (Goodall and Vorhaus, 2011, p. 16). However, based on our observations, many parents may need to be supported by teachers regarding how to effectively guide and supervise children’s L2 learning. As the majority of Chinese parents attend parent–teacher conferences (*cf.* Li, 2006), the schools and teachers may use these conferences as a venue to share with the parents their instructional practices, curriculum and philosophies germane to L2 learning and teaching in creative ways; specifically, for example, the traditional parent–teacher conference format can be changed into smaller group-based activities, where both stakeholders exchange ideas, beliefs and strategies in relaxed setting; the traditional format may also be supplemented with demonstration sessions or mini-workshops, where good practice in supporting the development of L2 proficiency is shared and discussed. Collaboration between parents at home and teachers in school will be more helpful in enabling students to engage with L2 learning (and learning in general) than parents and schools working separately.

Despite its contributions (both substantive and methodological) and practical implications, this study has two major limitations. Firstly, although to evaluate engagement most studies (including the present one) have employed self-reports (Zhou et al., 2020), which can bring many advantages^11^, research on engagement and similar psychological IDs will stand to gain from a combination of different data sources, such as standardised tests, teacher evaluation, classroom observation and in-depth interviews. The above suggestions are not just applicable to the dependent variable, but also to some key independent variables (e.g., L2 proficiency). Secondly, in terms of the study population, the samples were taken among secondary school students; a similar study conducted with university students or primary school students could possibly yield different results; due to the disparity in educational resources provision between rural and urban regions (Butler, 2015), a similar study targeting students in economically under-developed rural areas may generate different and interesting insights (*cf.* Wei and Hu, 2021). Further studies overcoming (some of) these limitations are needed to corroborate, modify, or falsify the tentative conclusions offered by the present study.

## Data Availability Statement

The raw data supporting the conclusions of this article will be made available by the authors, without undue reservation.

## Ethics Statement

The studies involving human participants were reviewed and approved by High School Affiliated to Southwest University. Written informed consent to participate in this study was provided by the participants’ legal guardian/next of kin.

## Author Contributions

JW: conceptualization, funding, in-depth data analysis, and original draft preparation. BY: data collection and preliminary data analysis. ZL: preliminary data analysis and original draft preparation. RW: conceptualization, funding, in-depth data analysis, original draft preparation, reviewing and editing, and supervision. Each of the authors listed has made a substantial, direct, intellectual contribution to this original research, and approved it for publication. All authors contributed to the article and approved the submitted version.

## Funding

The writing of this paper was supported by Jiangsu Education Department (Grant No. 2019SJA0437), Nanjing Xiaozhuang University (Grant No. 2019NXY34), and Xi’an Jiaotong-Liverpool University (the Research Enhancement Fund, REF-19-02-01).

## Conflict of Interest

The authors declare that the research was conducted in the absence of any commercial or financial relationships that could be construed as a potential conflict of interest.

## Publisher’s Note

All claims expressed in this article are solely those of the authors and do not necessarily represent those of their affiliated organizations, or those of the publisher, the editors and the reviewers. Any product that may be evaluated in this article, or claim that may be made by its manufacturer, is not guaranteed or endorsed by the publisher.

## Footnotes

^1^As will be further discussed below, L2 engagement is operationalised as a student’s score gained on a L2 engagement scale adapted from the UWES-S.

^2^The literature review blow focuses on (primarily) quantitative studies because of space constraints. Interesting empirical studies that are primarily qualitative in nature include Langhout and Mitchell (2008), Chiu (2021), and Fargo and Mastrangelo (2021). For example, Pineda-Báez et al.’s (2019) qualitative study examined the perceptions of students to determine factors that might reinforce or hinder student engagement. A total of 150 students from four public schools from three municipalities in Colombia took part in the study. Data were collected from group interviews and student texts. These researchers’ qualitative analysis identified two important factors affecting student engagement: teachers’ encouragement and a healthy school climate.

^3^Echoing the calls for stronger methodological rigour in applied linguistics research (Plonsky, 2013; Wei and Hu, 2019), our discussion of quantitative results focuses on effect size. Whenever possible, we examine relevant effect sizes from previous studies, and (re)interpret the results according to the effect size benchmarks proposed by Wei and Hu (2019): 0.005, 0.01, 0.02, and 0.09, respectively, represent the small/weak, typical (medium), large/strong, and very large/strong cut-offs for the effect size index *R*^2^; when *R*^2^ is un-squared, the corresponding benchmarks for the effect size index *r* are.07, 0.10, 0.14, and 0.30.

^4^Research at this level has taken place in Content and Language Integrated Learning (CLIL) settings mostly in Europe, and has alluded to a link between engagement and L2 proficiency. For instance, multilingual resources including L2 proficiency ‘are linked to the participation regime’ (Gajo et al., 2013, p. 289). What these authors termed as ‘participation regime’ largely overlaps with what we call ‘engagement’ with L2.

^5^This can be reflected in many instances of wording such as ‘significant difference’ and ‘significant predictor’ in the above-reviewed studies. This tendency ‘to drop the word *statistically* and use “significant difference” instead of “statistically significant difference” in research reports’ (Nassaji, 2012, p. 95) revealed the authors’ unfortunate subscription to the Top 1 most common misconception that ‘statistically significant means important’ (see Nassaji, 2012 for the other eight misconceptions).

^6^Only a very limited number of quantitative studies have examined the link between parental input and student engagement *with general learning*. For example, Stubbs and Maynard (2017) found that, in their sample of 523 post-secondary school students, parental input (operationalised as ‘family cohesion and adaptability’) explained 13.5% of the variance in student engagement.

^7^To be more specific, Butler (2015) utilised four items on a five-point Likert scale: (1) the frequency with which the parents helped their children in their studies in general; (2) their help with English study only; (3) their involvement in school activities; and (4) their information gathering through their networks. One of our two measures (namely frequency of parental coaching for L2 learning) largely overlaps with Butler’s (2015) variable. We decided not to adopt all of her measures because space constraints in our instrument, which was used for a larger project (see Wang et al., 2021), precluded the possibility of using more items to measure parental input.

^8^ https://www.wjx.cn/

^9^Aubrey et al. (2020) found that a lack of engagement could be attributed to causes such as ‘lack of vocabulary’ and being unable to say what the student wanted to ‘say quickly in English’ (p. 8). In other words, L2 proficiency level could affect engagement.

^10^Martin et al.’s (2020) finding that non-English speaking background statistically significantly correlated with negative engagement (*β* = 0.03) pointed to the possible link between L2 proficiency and engagement.

^11^Out of practical and other considerations, measures of self-rated proficiency can be used as acceptable indicators of actual proficiency (*cf.* Zhang et al., 2020) especially when objective ratings are not readily available. As early as in the 1990s, MacIntyre et al. (1997) confirmed the strong intercorrelation between scores for perceived L2 competence and actual L2 competence; most recently, Gullifer et al. (2021) demonstrated that students are generally able to accurately self-rate their L2 proficiency through subjective measures.
