# Supplementary material for: Exploring L2 Engagement: A Large-Scale Survey of Secondary School Students
Source: Front Psychol. 2022 Jun 14;13:868825. doi: 10.3389/fpsyg.2022.868825 (PMC9239970; doi:10.3389/fpsyg.2022.868825)
Supplement: Supplementary file 1 [file Data_Sheet_1.docx]

Appendix 1. Exploratory Factor Analysis of the L2 Engagement Scale

| Items | Factor 1 |
| --- | --- |
| 1. 学习英语时，我浑身有力而且干劲十足。(I feel strong and vigorous when I am studying English or going to English class.) | .878 |
| 1. 我沉浸在英语学习中。(I am immersed in my studies. ) | .874 |
| 9. 我对英语学习充满了热情。(I am enthusiastic about my English studies.) | .874 |
| 1. 全身心投入英语学习时，我感到很快乐。(I feel happy when I am studying English intensely.) | .863 |
| 11. 我发现我的英语学习目的明确，而且很有意义。(I find my English studies full of meaning and purpose.) | .847 |
| 8. 英语学习能激发我的灵感。(My English study inspires me.) | .843 |
| 14. 学习英语时，我心里只想着学习。(I get carried away when I am studying English.) | .832 |
| 15. 我难以放下手中的英语学习。(It is difficult to detach myself from my English studies.) | .820 |
| 10. 我因我的英语学习而感到自豪。(I am proud of my English studies.) | .817 |
| 2. 学习英语时，我感到精力充沛。(When I am doing my English work as a student, I feel bursting with energy.) | .816 |
| 5. 学习英语时，即使精神疲劳，我也能很快恢复。(I am very resilient, mentally, as far as my English studies are concerned.) | .795 |
| 13. 学习英语时，我感到时间过得很快。(Time flies when I am studying English.) | .794 |
| 12. 学习英语时，我会忘了周围的一切。(When I am studying English, I forget everything else around me.) | .790 |
| 1. 即使英语学习不顺利，我也毫不气馁，能够坚持不懈。(As far as my English studies are concerned I always persevere, even when things do not go well.) | .768 |
| 4. 我能持续学习英语很长时间，中间不需要休息。(I can continue English studying for very long periods at a time.) | .748 |
| 7. 我发现英语学习富有挑战性。(To me, my English studies are challenging.) | .741 |
| 1. 早晨一起床，我就乐意去学习英语。(When I get up in the morning, I feel like going to English class.) | .686 |

Appendix 2. First-order correlated factor structure of UWES-S scale measuring L2 engagement


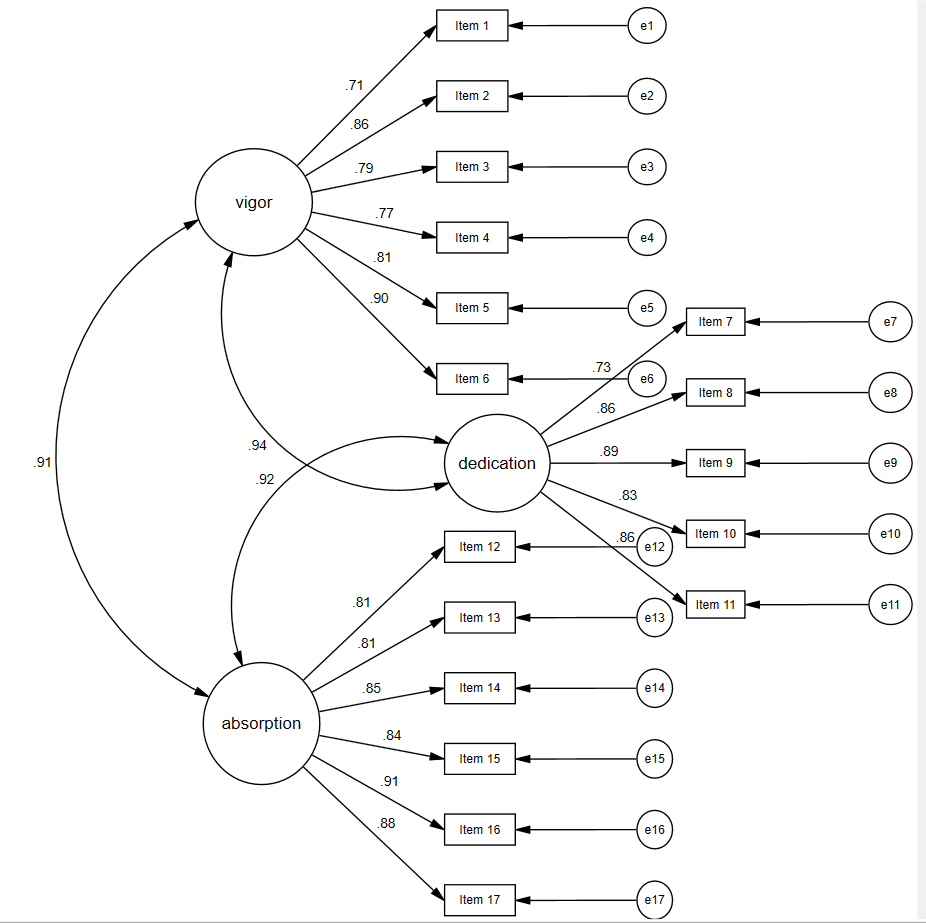


Appendix 3. Second-order factor structure of UWES-S scale measuring L2 engagement


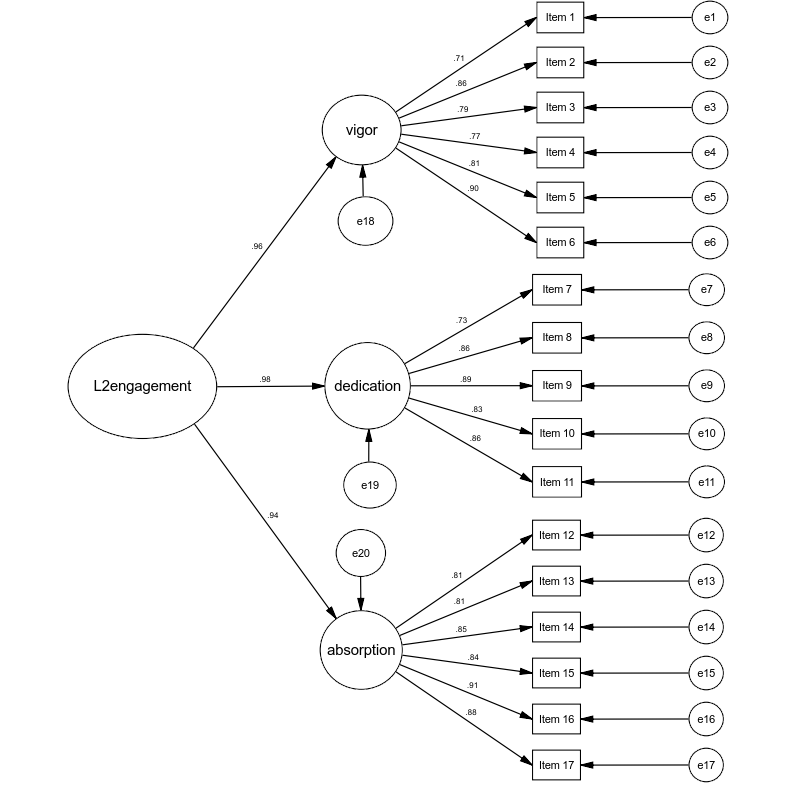


Appendix 4. Hierarchical Regression Predicting L2 Engagement: Results.

|  | | *B* | *SEB* | *β* | *p* |  |  |  |  | ***R^2^*** | ***△R^2^*** | ***△F*** |
| --- | --- | --- | --- | --- | --- | --- | --- | --- | --- | --- | --- | --- |
| **Model 1** | |  |  |  |  |  |  |  |  | **.100** | **.100** | **2301.092** |
|  | (Constant) | 2.532 | .030 |  | < .0005 |  |  |  |  |  |  |  |
|  | Parental attention | .389 | .008 | .317 | *<* .0005 |  |  |  |  |  |  |  |
| **Model 2** | |  |  |  |  |  |  |  |  | **.121** | **.020** | **475.567** |
|  | (Constant) | 2.510 | .030 |  | *<* .0005 |  |  |  |  |  |  |  |
|  | Parental attention | .312 | .009 | .254 | *<* .0005 |  |  |  |  |  |  |  |
|  | Parental coaching frequency | .160 | .007 | .156 | *<* .0005 |  |  |  |  |  |  |  |
| **Model 3** | |  |  |  |  |  |  |  |  | **.188** | **.067** | **1712.171** |
|  | (Constant) | 2.033 | .031 |  | *<* .0005 |  |  |  |  |  |  |  |
|  | Parental attention | .252 | .009 | .205 | *<* .0005 |  |  |  |  |  |  |  |
|  | Parental coaching frequency | .119 | .007 | .116 | *<* .0005 |  |  |  |  |  |  |  |
|  | Study time | .430 | .010 | .270 | *<* .0005 |  |  |  |  |  |  |  |
| **Model 4** | |  |  |  |  |  |  |  |  | **.190** | **.002** | **38.582** |
|  | (Constant) | 2.226 | .044 |  | *<* .0005 |  |  |  |  |  |  |  |
|  | Parental attention | .248 | .009 | .202 | *<* .0005 |  |  |  |  |  |  |  |
|  | Parental coaching frequency | .117 | .007 | .113 | *<* .0005 |  |  |  |  |  |  |  |
|  | Study time | .428 | .010 | .270 | *<* .0005 |  |  |  |  |  |  |  |
|  | Being only-child or not | -.099 | .016 | -.039 | *<* .0005 |  |  |  |  |  |  |  |
| **Model 5** | |  |  |  |  |  |  |  |  | **.194** | **.004** | **103.399** |
|  | (Constant) | 1.993 | .049 |  | *<* .0005 |  |  |  |  |  |  |  |
|  | Parental attention | .242 | .009 | .200 | *<* .0005 |  |  |  |  |  |  |  |
|  | Parental coaching frequency | .113 | .007 | .110 | *<* .0005 |  |  |  |  |  |  |  |
|  | Study time | .426 | .010 | .268 | *<* .0005 |  |  |  |  |  |  |  |
|  | Being only-child or not | -.072 | .016 | -.029 | *<* .0005 |  |  |  |  |  |  |  |
|  | Monthly family income | .062 | .006 | .065 | *<* .0005 |  |  |  |  |  |  |  |
| **Model 6** | |  |  |  |  |  |  |  |  | **.195** | **.002** | **41.400** |
|  | (Constant) | 1.902 | 0.51 |  | *<* .0005 |  |  |  |  |  |  |  |
|  | Parental attention | .239 | .009 | .194 | *<* .0005 |  |  |  |  |  |  |  |
|  | Parental coaching frequency | .105 | .007 | .101 | *<* .0005 |  |  |  |  |  |  |  |
|  | Study time | .425 | .010 | .268 | *<* .0005 |  |  |  |  |  |  |  |
|  | Being only-child or not | -.039 | .017 | -.016 | .019 |  |  |  |  |  |  |  |
|  | Monthly family income | .046 | .007 | .048 | *<* .0005 |  |  |  |  |  |  |  |
|  | Mother’s education qualification | .052 | .008 | .048 | *<* .0005 |  |  |  |  |  |  |  |
| **Model 7** | |  |  |  |  |  |  |  |  | **.196** | **.001** | **13.985** |
|  | (Constant) | 1.870 | .052 |  | *<* .0005 |  |  |  |  |  |  |  |
|  | Parental attention | .238 | .009 | .194 | *<* .0005 |  |  |  |  |  |  |  |
|  | Parental coaching frequency | .103 | .007 | .100 | *<* .0005 |  |  |  |  |  |  |  |
|  | Study time | .425 | .010 | .267 | *<* .0005 |  |  |  |  |  |  |  |
|  | Being only-child or not | -.032 | .017 | -.013 | .055 |  |  |  |  |  |  |  |
|  | Monthly family income | .041 | .007 | .042 | *<* .0005 |  |  |  |  |  |  |  |
|  | Mother’s education qualification | .030 | .010 | .028 | .002 |  |  |  |  |  |  |  |
|  | Father’s education qualification | .038 | .010 | .034 | *<* .0005 |  |  |  |  |  |  |  |
| **Model 8** | |  |  |  |  |  |  |  |  | **.413** | **.217** | **7622.713** |
|  | (Constant) | 1.207 | .045 |  | *<* .0005 |  |  |  |  |  |  |  |
|  | Parental attention | .163 | .007 | .132 | *<* .0005 |  |  |  |  |  |  |  |
|  | Parental coaching frequency | .096 | .006 | .093 | *<* .0005 |  |  |  |  |  |  |  |
|  | Study time | .261 | .009 | .164 | *<* .0005 |  |  |  |  |  |  |  |
|  | Being only-child or not | -.003 | .014 | -.001 | .858 |  |  |  |  |  |  |  |
|  | Monthly family income | -.013 | .006 | -.013 | *<* .0005 |  |  |  |  |  |  |  |
|  | Mother’s education qualification | -.017 | .009 | -.016 | *<* .0005 |  |  |  |  |  |  |  |
|  | Father’s education qualification | -.010 | .009 | -.009 | *<* .0005 |  |  |  |  |  |  |  |
|  | L2 proficiency | .562 | .006 | .506 | *<* .0005 |  |  |  |  |  |  |  |
